# COVID-19-Related Quarantine Effect on Dietary Habits in a Northern Italian Rural Population: Data from the Brisighella Heart Study

**DOI:** 10.3390/nu13020309

**Published:** 2021-01-22

**Authors:** Arrigo F. G. Cicero, Federica Fogacci, Marina Giovannini, Martina Mezzadri, Elisa Grandi, Claudio Borghi, the Brisighella Heart Study Group

**Affiliations:** 1 Hypertension and Atherosclerosis Research Group, Medical and Surgical Sciences Department, Sant’Orsola-Malpighi University Hospital, 40138 Bologna, Italy; federica.fogacci@studio.unibo.it (F.F.); marina.giovannini3@unibo.it (M.G.); martina.mezzadri@studio.unibo.it (M.M.); elisa.grandi@unibo.it (E.G.); claudio.borghi@unibo.it (C.B.); 2 IRCCS Azienda Ospedaliero-Universitaria di Bologna, 40138 Bologna, Italy

**Keywords:** COVID-19, quarantine, lockdown, epidemiology, diet, lifestyle

## Abstract

North of Italy was severely hit by the COVID-19 (Coronavirus disease 19) pandemic. This induced the government to adopt severely restrictive measures to reduce the contagion risk, forcing most of the population to stop working and from leisure activities, and to remain at home for several weeks. Our study aimed to evaluate the effect of COVID-related quarantine on smoking and dietary habits of a well-characterized northern Italian rural population. For this purpose, while lockdown restrictions were in place (February–April 2020), 359 subjects from the Brisighella Heart Study cohort underwent a phone interview about their lifestyle habit changes during COVID-19-related quarantine. Quarantine did not significantly modify smoking habit nor body mass index. Subjects significantly increased daily carbohydrates consumption, all fresh vegetables, healthy vegetable oils, milk and yogurt, alcoholic drinks, sugars and sweets, and coffee. The weekly consumption of low-fat meat, cured meat other than ham, cheeses, eggs, nuts and mixed seed oils significantly increased, while the weekly intake of fish, mussels, and legumes significantly decreased during lockdown. The Dietary Quality Index was reduced from 42.4 ± 4.1 to 37.8 ± 4.7 (*p* < 0.03). In accordance with our findings, COVID-19-related quarantine might worsen the quality of diet, also leading to an increased intake of almost all food categories.

## 1. Introduction

Recently, the North of Italy has been severely hit by COVID-19 pandemic [1,2]. This induced the Italian government to adopt severely restrictive measures in order to reduce the risk of contagion, forcing most of the population to stop working and from leisure activities, and to remain at home for several weeks. Indeed, quarantine was the most effective method to reduce the infection risk [3]. Even though there are some dietary guidelines for COVID-19 patients [4,5], less it is known about subjects in quarantine, mainly because of lack of data on dietary intake in the general population.

The Food and Agriculture Organization (FAO) recommended not to eat food as a comfort and limit calories preferring fruits and vegetables [6]; however, it is still unknown how quarantine influenced dietary habits of isolated people in different countries. Some preliminary data are available from China [7], Spain [8] and Poland [9,10,11]. Some data are also available on children [12]. Overall, the change in food consumption seems to be largely different in different countries, partly related to the different restriction measures decided by the different governments to confront the COVID-19 pandemic [13]. As a matter of fact, the promotion of a healthy lifestyle is difficult in the general population and the maintenance of a high level of attitude toward healthy recommendations can be even more difficult in times of high stress [14]. Data are needed to attenuate the development of false myths on the association between diet and COVID-19 [15], but also to more clearly define the consequences of COVID-related quarantine on the life-styles of the general population.

The aim of our study was to evaluate the effect of COVID-related quarantine on smoking and dietary habits of a northern Italian rural population. In particular, the selection of subjects from a well-characterized cohort could help to reduce the heterogeneity of the answers and lead to more reliable information, hopefully useful in writing and supporting focused guidelines to prevent negative dietary changes during quarantine.

## 2. Materials and Methods

The Brisighella Heart Study (BHS) is a longitudinal population study with data collection since 1972. It is a randomized sample and is representative of the entire population of Brisighella, a rural North-Italian village. At the baseline, the BHS enrolled 2939 Caucasian adult volunteers (1491 men and 1448 women) in primary prevention for cardiovascular diseases. For every subject, we recorded a detailed personal history (mainly focused on lifestyle and dietary habits, smoking status and pharmacological treatments), anthropometric data, resting blood pressure and heart rate, the results of standard hematochemistry and of a 12-lead electrocardiogram (Minnesota-coded) [16]. In particular, over time, the BHS cohort dietary habits has been regularly assessed by the use of the Dietary Quality Index (DQI), a validated tool providing information on the usual food intake of 18 food items, grouped in three food categories [17]. Based on the questionnaire results, an estimation of the energy intake has also been calculated.

The study has been carried out in agreement with the Declaration of Helsinki. Its protocol—previously described elsewhere—was approved by the institutional ethical board of the University Hospital of Bologna (Code: BrixFollow-up_1972–2024) and all involved a signed informed consent form at entry from the subjects [18].

**Figure 1:**
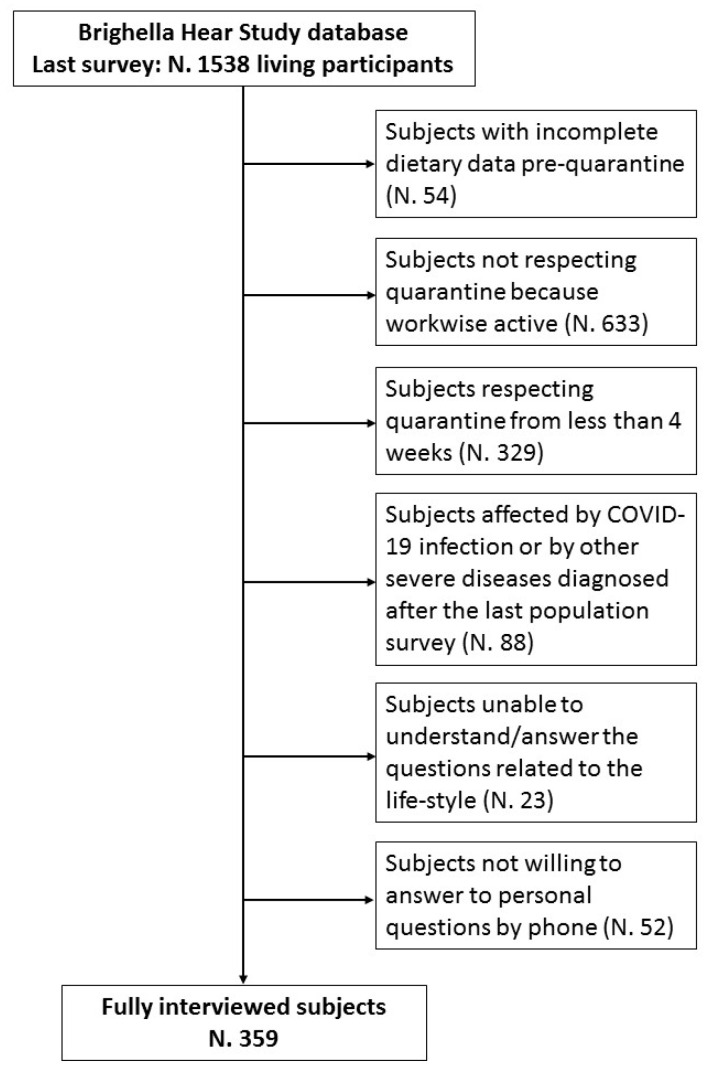
Flow-chart resuming the selection criteria for the sub-study analysis.

For the purpose of this sub-study, while the lockdown restrictions were in place (February–April 2020), subjects underwent a phone interview about their lifestyle habit changes during the COVID-19-related quarantine. Participants were withdrawn if they were workers not in quarantine (e.g., healthcare personnel, police forces, military personnel and farmers), if they were in quarantine from less than four weeks, or were affected by COVID-19 infection or other severe diseases diagnosed after the last population survey. We also excluded subjects who could not answer questions regarding their lifestyle, or who did not wish to answer personal questions over the phone (Figure 1).

The DQI questionnaire was administered by trained personnel to 359 adult-elderly subjects (men: 156, women: 203; mean age: 64.6 ± 13.3 years).

The main categories of food investigated by intake frequency were resumed in Table 1. Combined dishes as a standard Pizza were considered as a portion of bread-like product and a portion of cheese.

After the quarantine, we recalled and again administered the DQI to those subjects who had previously changed their dietary habits and who were no longer in quarantine, in order to check eventual further dietary changes. The change in energy intakes from different diet components was also estimated.

A full descriptive analysis was carried out for all the considered parameters. A Kolmogorov-Smirnov normality test was performed for the continuous variables. Quantitative data were compared by the t-test for paired samples or by the Wilcoxon rank test. The analyses were repeated by gender, education level, age class and by the median quarantine duration at the time of the interview. The study power was based on the basis of the consumption of sugars and sweets, as markers of un-healthy diet. All tests were carried out using SPSS 24.0 for Windows (IBM Corporation, Armonk, NY, USA). A significance level of 0.05 was considered to be valid for every test.

## 3. Results

Considering the baseline sugars and sweet consumption, the change observed and the sample size, the study was adequately powered (100%).

Quarantine neither significantly modified smoking habit (2.2% reduced their habit, 1.7% increased smoking) nor body mass index (BMI) (26.6 ± 4.7 vs. 26.9 ± 4.5 kg/m^2^, *p* = 0.361) of respondents. The 19.2% of the subjects began to take a dietary supplement during lockdown restrictions. The most frequently used dietary supplements were lipid-lowering nutraceuticals (24.5%), multivitamin/mineral supplements (21.9%), vitamin C alone (11.5%) and magnesium alone (11.0%).

Self-perceived dietary changes reported by the interviewed subjects were the following: (A) no change (50%), (B) overall increased quantity of food assumed (32%), (C) overall decreased quantity of food assumed (6.4%), (D) main increase in sweets intake (4.2%), (E) improved quality of diet composition (3.4%), (F) main increase in complex carbohydrates (bread, pizza, pasta, rice; 2.2%), and (G) decreased quality of diet composition (0.9%).

Among the subjects who changed their dietary habits during lockdown restrictions (N = 178), at the time of the interview, three volunteers were yet in quarantine because dwelling with patients infected with severe acute respiratory syndrome-related coronavirus-2 (SARS-CoV-2), and five were lost to follow-up.

**Figure 2:**
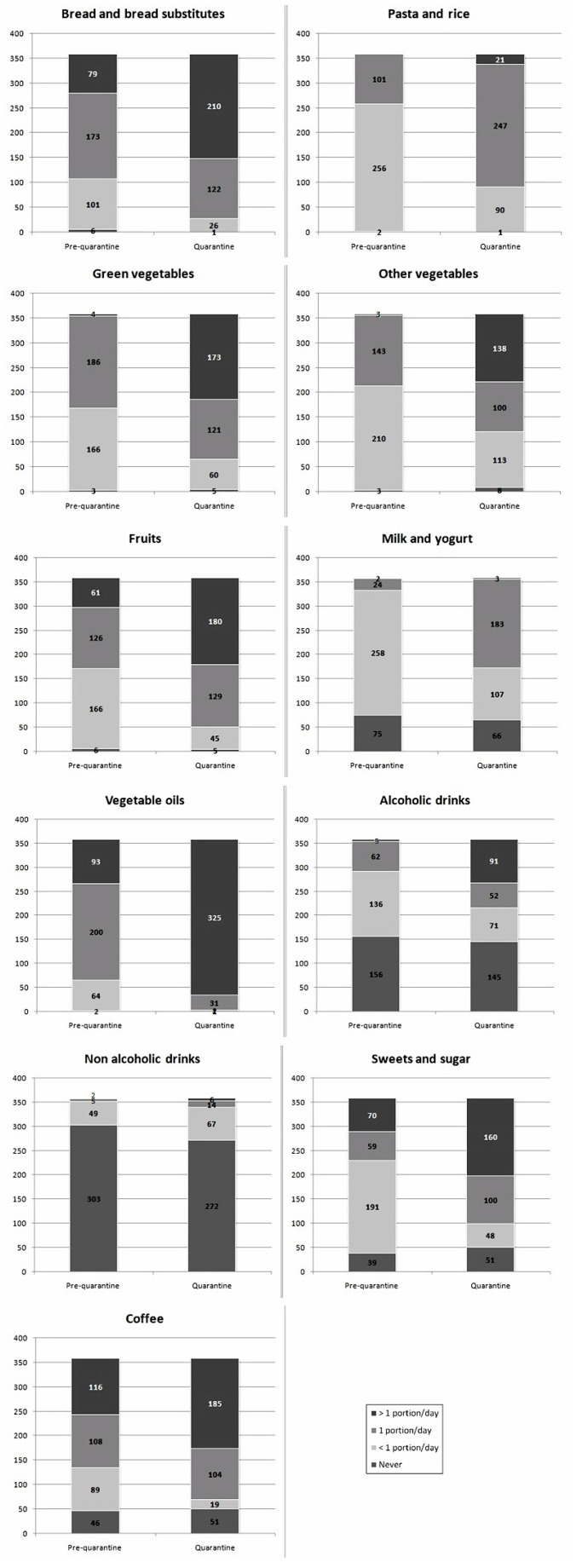
Changes in single food groups intake during quarantine (portions/day).

**Figure 3:**
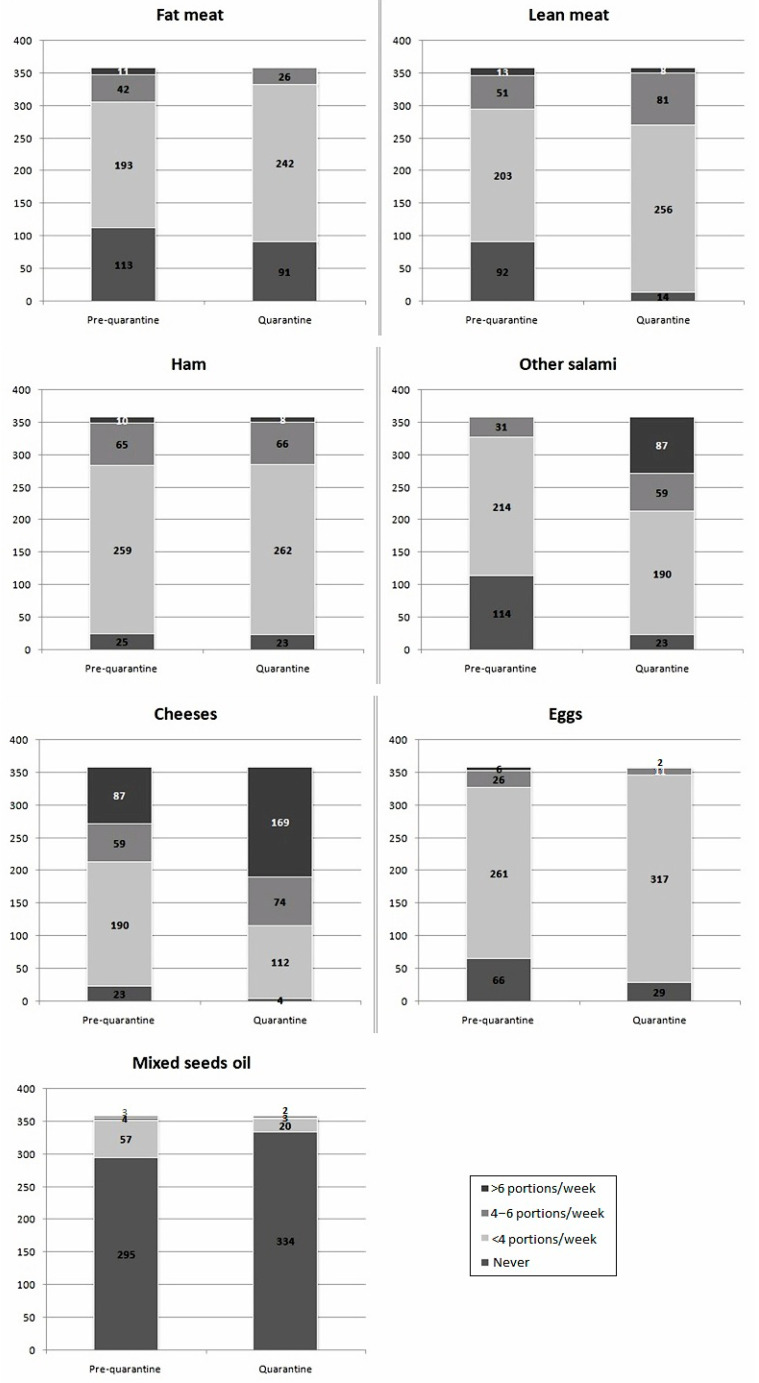
Changes in single food groups intake during quarantine (portions/weeks).

**Figure 4:**
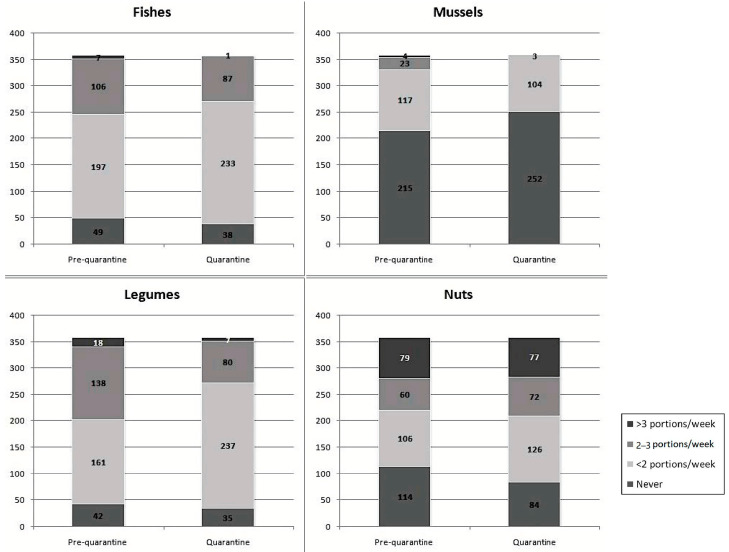
Changes in fishes, mussels, legumes and nuts intake during quarantine (portions/week).

Respondents’ main dietary changes are presented in Figure 2, Figure 3 and Figure 4.

During lockdown, subjects significantly increased the daily consumption of bread and bread-like products (Z = −10.959), pasta and rice (Z = −15.415), green vegetable (Z = −13.453) and other vegetables (Z = −12.078), healthy vegetable oils (Z = −11.422), fruits (Z = −10.171), milk and yogurt (Z = −13.839), alcoholic drinks (Z = −9.910), simple sugars and sweets (Z = −8.955), and coffee (Z = −4.783) (*p* < 0.001 always). Only daily intake of non-alcoholic drinks was unchanged (Z = −0.282, *p* = 0.778). Among foods consumed from 0–6 or more times per week, the consumption of low-fat meat (Z = −6.025), cured meats other than ham (Z = −4.365), cheeses (Z = −7.415), eggs (Z = −2.422, *p* = 0.015), and mixed seed oils (Z = −3.928) significantly increased during lockdown restrictions (*p* < 0.001 for all comparisons, unless otherwise specified). On the contrary, the intake of non-cured fat meat (Z = −0.058, *p* = 0.954) and ham (Z = −0.208, *p* = 0.835) did not significantly change. Among foods consumed from 0–3 or more times per week, during quarantine, the consumption of fish (Z = −4.011), mussels and shellfish (Z = −7.072,), and legumes (Z = −4.294), significantly decreased (*p* < 0.001 always), while the consumption of nuts (Z = −2.037, *p* = 0.042) slightly increased. Water consumption did not vary (Z = −1.816, *p* = 0.69). The mean DQI decreased from 42.4 ± 4.1 to 37.8 ± 4.7 (*p* = 0.011).

The mean energy intake was 2568 ± 322 kcal before the lockdown and 2739 ± 442 kcal during the quarantine (Z = −11.231, *p* < 0.001).

The changes in main diet components are presented in Table 2: during quarantine the interviewed subjects significantly increased the consumption of simple sugars, added fats and alcohol, while overall increasing the carbohydrates and fat intake.

By repeating the analysis by gender, education level, and age class, we confirmed the reported results. When repeating the analyses by the median quarantine duration at the time of the interview (43 days, 95% 21 to 78), we also did not observe specific changes in subjects who had just begun the quarantine, with the ones tolerating it for a longer time.

In comparison with the quarantine period, the remaining group (men: 73, women: 97; mean age: 65.1 ± 11.9 years) did not change dietary habits, beyond significantly increasing intake of water (Z = −2.365, *p* = 0.018) and alcoholic drinks (Z = −8.746, *p* = 0.033). The mean DQI also remained unchanged (*p* = 0.134).

## 4. Discussion

In accordance with FAO recommendations, the maintenance of a healthy diet is an important part of supporting the immune system since no single food or dietary supplement are known to prevent the SARS-CoV-2 infection [5]. Thus, it is important to check the change of lifestyle during prolonged quarantine. Furthermore, subjects who seem to be more susceptible to SARS-CoV-2 are those with hypertension, obesity and type 2 diabetes [19], which are lifestyle related cardiovascular risk factors that could be negatively influenced by quarantine.

Quarantine is, per se, irreducibly associated with an increased level of some cardiovascular risk factors (e.g., sedentariness and psychological stress) [20]. Even though quarantine protects people from contagion, our findings suggest that, in the absence of large-scale nutrition education programs, the population might choose a lower quality diet. This could then favor the development or aggravation of conditions increasing the health risk related to COVID-19. In fact, we observed that the interviewed subjects significantly increased the daily consumption of bread and substitutes, pasta and rice, green vegetable and other vegetables, healthy vegetable oils, fruits, milk and yogurt, alcoholic drinks, sugars and sweets, and coffee. Furthermore, the consumption of lean meat, salamis other than ham, cheeses, eggs, nuts and mixed seed oils significantly increased. On the contrary, the consumption of fish, mussels, and legumes significantly decreased during lockdown, and the DQI was significantly reduced. The increase in food intake is very similar to what was observed in another cohort in Northern Italy [21]. The energy intake change is also similar to what observed in another Mediterranean country strongly affected by the COVID-19 pandemic, Spain, where the energy intake increased by around 30% during the lockdown period [22].

There are many hypotheses that could try to explain the observed results. People could find psychological support in the intake of food. However, the quarantine has also provided more free time to dedicate to cooking and eating, thus making it easier to overeat. The limited availability of access to food shops (because of the closing of smaller shops, and reduced and slowed deliveries from industries) could have induced the people to accumulate a large amount of food at home, increasing its ready availability. The increase in water and alcoholic drinks could be, on the contrary, related to the early summer heat.

The apparent discrepancy between energy intake increased and the lack of BMI increase could be explained, in part, by the fact that about a half of the interviewed subjects did not significantly change their food intake, and in part from the fact that some of the subjects were interviewed after a relatively short time from the beginning of the quarantine. The DQI score decreased even when the subjects also increased the intake of protective foods such as fish and vegetables, including legumes and fruits. However, the increased intake of simple sugars, added fats, and alcohol decreased the overall quality of diets.

Comparing the subjects’ interviews after the end of the lockdown restrictions, we observed that people seemed to keep the habits acquired during the quarantine, both from a quantitative and qualitative point of view. However, the comparison of the periods “during” and “after” the lockdown was also complex, since in Italy the situation is continuously in evolving (both from a viral and a legal point of view), so that elderly people are more likely to be sequestered than younger ones and some categories of workers are yet to be limited in their standard pre-COVID-19 pandemic activities. The lack of reverse change of dietary habits after the lockdown period should, however, also be partially explained by the short time after the quarantine and the second interview. Anyway, the lack of improvement of dietary habits after quarantine could show the need for further educational campaigns.

Our observation is limited by not considering the metabolic effects of the dietary changes observed during the lockdown restrictions. However, our aim was to evaluate the extent of dietary habit changes that could be potentially dangerous in the long-term.

The citizens of Brisighella are periodically solicited by educational messages on healthy lifestyle as part of the BHS; then, we might have underestimated the impact of quarantine on dietary habits of our respondents [18]. Moreover, our observations could not be directly inferred for other populations, as suggested by the results of other studies conducted in different contexts [23].

Overall, our findings suggest a higher need to focus educational campaigns on dietary advice during the COVID-19 pandemic in order to prevent dangerous dietary changes and to recover a more healthy diet pattern after quarantine periods. Physical activity promotion could also be an effective tool to partially counterbalance the negative impact of food intake modification [24].

## 5. Conclusions

COVID-19-related quarantine might worsen the quality of diet, also leading to an increased intake of almost all categories of food. In particular, our results show a trend towards decreasing diet quality that could flag future health problems. For this reason, the promotion of more nutritional awareness is necessary.

## Figures and Tables

**Table 1:** Investigated food categories by intake frequency.

Daily Intake	Frequent Week Intake (0–6 Portion per Week)	Less Frequent Week Intake (0–3 Portion per Week)
- Bread and bread-like products (i.e., dinner rolls, flat breads) - Pasta and rice - Green vegetables and other vegetables - Healthy vegetable oils (i.e., extra-virgin olive oil, corn oil) - Fruits - Milk and yogurt - Non-alcoholic drinks - Alcoholic drinks - Sugars and sweets (including cookies, croissants and sweet rolls) - Coffee - Water	- Low-fat meat (i.e., skinless poultry, red meat cooked after visible fat is trimmed off) - Non-cured fat meat (i.e., red meat cooked with fat) - Cured meats other than ham - Ham - Cheeses - Eggs - Mixed seed oil	- Fish - Mussels and shellfish - Legumes - Nuts

**Table 2:** Percentage of total energy derived from the main diet components of the interviewed subjects.

Diet Components	Pre-Quarantine Intake (%)	Intake during Quarantine (%)	*p*
Total carbohydrates	49.3 ± 4.6	52.6 ± 6.5	0.048
- Simple sugars	3.1 ± 0.9	4.6 ± 1.1	0.002
Total fats	28.1 ± 3.2	31.4 ± 2.9	0.047
- Added fats	3.9 ± 1.1	4.3 ± 1.2	0.021
Proteins	16.2 ± 2.6	10.1 ± 2.7	0.003
Alcohol	2.9 ± 0.6	4.9 ± 1.0	0.002

## Data Availability

The data that support the findings of this study are available from the University of Bologna. Data are available from the author with the permission of the University of Bologna.

## Availability of Data and Material

Original data will be made available on request to arrigo.cicero@unibo.it.

## References

[1] Ferrari R., Maggioni A.P., Tavazzi L., Rapezzi C. (2020). The battle against COVID-19: Mortality in Italy. Eur. Heart J..

[2] Mancusi C., Grassi G., Borghi C., Carugo S., Fallo F., Ferri C., Giannattasio C., Grassi D., Letizia C., Minuz P. (2020). Determinants of healing among patients with coronavirus disease 2019: The results of the SARS-RAS study of the Italian Society of Hypertension. J. Hypertens..

[3] Nussbaumer-Streit B., Mayr V., Dobrescu A.I., Chapman A., Persad E., Klerings I., Wagner G., Siebert U., Christof C., Zachariah C. (2020). Quarantine alone or in combination with other public health measures to control COVID-19: A rapid review. Cochrane Database Syst. Rev..

[4] Fernández-Quintela A., Milton-Laskibar I., Trepiana J., Gómez-Zorita S., Kajarabille N., Léniz A., González M., Portillo M.P. (2020). Key Aspects in Nutritional Management of COVID-19 Patients. J. Clin. Med..

[5] Azzolino D.M., Saporiti E., Proietti M., Cesari M. (2020). Nutritional Considerations in Frail Older Patients with COVID-19. J. Nutr. Health Aging.

[6] FAO Maintaining a Healthy Diet during the COVID-19 Pandemic. http://www.fao.org/3/ca8380en/CA8380EN.pdf.

[7] Wang X., Lei S.M., Le S., Yang Y., Zhang B., Yao W., Gao Z., Cheng S. (2020). Bidirectional Influence of the COVID-19 Pandemic Lockdowns on Health Behaviors and Quality of Life among Chinese Adults. Int. J. Environ. Res. Public Health.

[8] Ruiz-Roso M.B., Knott-Torcal C., Matilla-Escalante D.C., Garcimartín A., Sampedro-Nuñez M.A., Dávalos A., Marazuela M. (2020). COVID-19 Lockdown and Changes of the Dietary Pattern and Physical Activity Habits in a Cohort of Patients with Type 2 Diabetes Mellitus. Nutrients.

[9] Górnicka M., Drywień M.E., Zielinska M.A., Hamułka J. (2020). Dietary and Lifestyle Changes During COVID-19 and the Subsequent Lockdowns among Polish Adults: A Cross-Sectional Online Survey PLifeCOVID-19 Study. Nutrients.

[10] Raciborski F., Jankowski M., Gujski M., Pinkas J., Samel-Kowalik P., Zaczyński A., Pańkowski I., Rakocy K., Wierzba W. (2020). Prevention of SARS-CoV-2 Infection Among Police Officers in Poland—Implications for Public Health Policies. Int. J. Environ. Res. Public Health.

[11] Sidor A., Rzymski P. (2020). Dietary Choices and Habits during COVID-19 Lockdown: Experience from Poland. Nutrients.

[12] Głąbska D., Skolmowska D., Guzek D. (2020). Population-Based Study of the Changes in the Food Choice Determinants of Secondary School Students: Polish Adolescents’ COVID-19 Experience (PLACE-19) Study. Nutrients.

[13] Murphy B., Benson T., McCloat A., Mooney E., Elliott C., Dean M., Lavelle F. (2021). Changes in Consumers’ Food Practices during the COVID-19 Lockdown, Implications for Diet Quality and the Food System: A Cross-Continental Comparison. Nutrients.

[14] Piepoli M.F., Hoes A.W., Agewall S., Albus C., Brotons C., Catapano A.L., Cooney M.T., Corrà U., Cosyns B., Deaton C. (2016). 2016 European Guidelines on cardiovascular disease prevention in clinical practice: The Sixth Joint Task Force of the European Society of Cardiology and Other Societies on Cardiovascular Disease Prevention in Clinical Practice (constituted by representatives of 10 societies and by invited experts) Developed with the special contribution of the European Association for Cardiovascular Prevention & Rehabilitation (EACPR). Atherosclerosis.

[15] El Ghoch M., Valerio A. (2020). Let food be the medicine, but not for coronavirus: Nutrition and food science, telling myths from facts. J. Popul. Ther. Clin. Pharmacol..

[16] Cicero A.F., Rosticci M., Tocci G., Bacchelli S., Urso R., D’Addato S., Borghi C. (2015). Serum uric acid and other short-term predictors of electrocardiographic alterations in the Brisighella Heart Study cohort. Eur. J. Intern. Med..

[17] Cicero A.F., Fogacci F., Desideri G., Grandi E., Rizzoli E., D’Addato S., Borghi C. (2019). Arterial Stiffness, Sugar-Sweetened Beverages and Fruits Intake in a Rural Population Sample: Data from the Brisighella Heart Study. Nutrients.

[18] Cicero A.F., Fogacci F., Grandi E., Rizzoli E., Bove M., D’Addato S., Borghi C. (2020). Prevalent Seasoning and Cooking Fats, Arterial Stiffness and Blood Lipid Pattern in a Rural Population Sample: Data from the Brisighella Heart Study. Nutrients.

[19] Richardson S., Hirsch J.S., Narasimhan M., Crawford J.M., McGinn T., Davidson K.W., Barnaby D.P., Becker L.B., Chelico J.D., Cohen S.L. (2020). Presenting Characteristics, Comorbidities, and Outcomes Among 5700 Patients Hospitalized with COVID-19 in the New York City Area. JAMA.

[20] Mattioli A.V., Ballerini Puviani M., Nasi M., Farinetti A. (2020). COVID-19 pandemic: The effects of quarantine on cardiovascular risk. Eur. J. Clin. Nutr..

[21] Cancello R., Soranna D., Zambra G., Zambon A., Invitti C. (2020). Determinants of the Lifestyle Changes during COVID-19 Pandemic in the Residents of Northern Italy. Int. J. Environ. Res. Public Health.

[22] Batlle-Bayer L., Aldaco R., Bala A., Puig R., Laso J., Margallo M., Vázquez-Rowe I., Antó J.M., Fullana-I-Palmer P. (2020). Environmental and nutritional impacts of dietary changes in Spain during the COVID-19 lockdown. Sci. Total Environ..

[23] Haddad C., Zakhour M., Bou Kheir M., Haddad R., Al Hachach M., Sacre H., Salameh P. (2020). Association between eating behavior and quarantine/confinement stressors during the coronavirus disease 2019 outbreak. J. Eat Disord..

[24] Violant-Holz V., Gallego-Jiménez M.G., González-González C.S., Muñoz-Violant S., Rodríguez M.J., Sansano-Nadal O., Guerra-Balic M. (2020). Psychological Health and Physical Activity Levels during the COVID-19 Pandemic: A Systematic Review. Int. J. Environ. Res. Public Health.

